# Man an Object of Zoology

**Published:** 1887-08

**Authors:** S. W. Stiles

**Affiliations:** Georgia


### MAN AN OBJECT OF ZOOLOGY.

BY S. W. STILES, M.D., GEORGIA.

Having a longing desire to know something of the *genus homo*, I have devoted many years in studying the subject and culled this paper from many of our most noted anatomists and zoologists and submit this, the result of my researches, without further comment.

The natural history of man, in its most comprehensive sense, constitutes a subject of immense extent, and of endless variety. In a complete history of man, it would be necessary to relate the phenomena of his first production, to examine his anatomical structure, his bodily and intellectual functions, his propensities and feelings, his diseases, and to pursue his progress from the time of birth to the grave. To write such a history of our species would demand a familiar acquaintance with nearly the whole circle of human knowledge, and a combination of the most opposite pursuits and talents. The anatomist and physiologist unfold the construction, and uses of the corporeal mechanism, the surgeon and physician describes its diseases, while the metaphysician and moralist employ themselves with those functions which constitute the mind, and with the moral sentiment. Man in society, his progress in various countries and ages of the world, his multiplication and extension, are the province of the historian and political economist. I desire to consider man as an object of zoology only, to describe him in the future as a subject of the animal kingdom.

What climates, what degrees of heat and cold can he bear? How is he able to endure all the diversified external influences of such various abodes? Is he indebted for this privilege to the strength, flexibility of his organization, or to his mental functions, his reason, and the arts, which he has thence derived? Is he a species broadly and clearly distinguished from all others, or is he specifically allied to the orang-outang and other monkeys? What are his corporeal, what his mental distinctions? Are the latter different in kind, or only superior in degree, to the higher order of animals ? Is there one species of men only, or are there many distinct ones ? What country did they first inhabit and what was the appearance of the original man ? Did he go erect, or on all fours? Was he a Patagonian, or an Esquimaux, a Negro, or a Georgian ? Such are the questions that claim our attention in a zoological survey of the human species. To suppose that it is in my power to furnish satisfactory replies, would be a degree of presumption which it is hardly necessary for me to disclaim.

The natural history of man is yet in its infancy, therefore a complete view of the subject could not be attempted. That the Negro is more like a monkey than the European cannot be denied, as a general observation, and as the monkey race approach the nearest to man in structure, and actions, and their forms are so much like the human, we must compare them to man, in order to find out the specific character of the latter and we must institute this comparison, particularly with those called orang-outangs. All the simiæ, and the lemurs likewise, are quadrumanous; that is, they possess opposable members, or thumbs on the hind as well as on the fore-limbs; they have perfect clavicles, perfect supination, and pronation of the fore-arm; long and flexible fingers and toes; hence they have the power of imitating many human actions, hence, too, they are excellent climbers. On the other hand, they cannot easily stand or walk erect, because the f ot rests on its outer edge, the heel does not touch the ground, and the narrowness of the pelvis renders the trunk unsteady. They resemble man in the general form of the cranium and in the configurations of the brain. The face is turned forwards; the optic axes are parallel, the orbits complete, and separate from the temporal fossæ; the nose is flat, and has a single triangular os nasi.

[To be Continued.]

